# West Virginia State Dental Society

**Published:** 1892-11

**Authors:** 


### West Virginia State Dental Society.

The first annual meeting of the West Virginia State Dental Society was held at Wheeling, October 5, 1892.

The officers elected for the ensuing year were as follows:

President—Dr. J. N. Mahan, Charleston; Vice-President—Dr. R. W. Tener, Wheeling; Treasurer—Dr. H. K. Jones, Parkersburg; Secretary—Dr. G. I. Keener, Morgantown.

