# A Correction

**Published:** 1892-12

**Authors:** 


### EDITORIAL.

### A Correction.

THE WORLD'S COLUMBIAN DENTAL CONGRESS.

The list of officers for the Columbian Dental Congress published in the November No. of the Register, was defective, owing to the omission of the names of Drs. A. W. Harlan, George J. Friedrichs, Louis Ottofy, Ralph Dillon, and John S. Marshall.

We publish below a complete list, which is as follows:

President, Dr. L. D. Shepard, of Boston, Mass.

Vice Presidents, Drs. W. W. H. Thackston, Farmville, Va.; Henry W. Morgan, Nashville, Tenn; A. L. Northrop, New York City, N. Y.; W. W. Allport, Chicago, Ill.; W. O. Kulp, Davenport, Iowa; C. S. Stockton, Newark, N. J.; E. T. Darby, Philadelphia, Pa.; H. J. McKellops, St. Louis, Mo.; J. H. Hatch, San Francisco, Cal.; J. B. Patrick, Charleston, S. C.; and John C. Storey, Dallas, Texas.

Secretary General, Dr. A. W. Harlan, Chicago, Ill.

Assistant Secretaries, Drs. Geo. J. Friedrichs, New Orleans, La.; Louis Ottofy, Chicago, Ill.; and Ralph Dillon, Chicago, Ill.

Treasurer, Dr. John S. Marshall, Chicago, Ill.

